# A Synthetic Cumate-Inducible Promoter for Graded and Homogenous Gene Expression in Pseudomonas aeruginosa

**DOI:** 10.1128/aem.00211-23

**Published:** 2023-05-18

**Authors:** Alexander Klotz, Andreas Kaczmarczyk, Urs Jenal

**Affiliations:** a Biozentrum, University of Basel, Basel, Switzerland; Danmarks Tekniske Universitet The Novo Nordisk Foundation Center for Biosustainability

**Keywords:** genetic tools, inducible promoter, *Pseudomonas aeruginosa*, synthetic biology

## Abstract

Inducible gene expression systems are powerful genetic tools to study bacterial physiology, probing essential and toxic gene functions, gene dosage effects, and overexpression phenotypes. For the opportunistic human pathogen Pseudomonas aeruginosa, dedicated inducible gene expression systems are scarce. In the current study, we developed a minimal synthetic 4-isopropylbenzoic acid (cumate)-inducible promoter, called P_QJ_, that is tunable over several orders of magnitude. This was achieved by combining semirandomized housekeeping promoter libraries and control elements from the Pseudomonas putida strain F1 *cym*/*cmt* system with powerful fluorescence-activated cell sorting (FACS) to select functionally optimized variants. Using flow cytometry and live-cell fluorescence microscopy, we demonstrate that P_QJ_ responds rapidly and homogenously to the inducer cumate in a graded manner at the single-cell level. P_QJ_ and cumate are orthogonal to the frequently used isopropyl β-d-thiogalactopyranoside (IPTG)-regulated *lacI*^q^-P_tac_ expression system. The modular design of the cumate-inducible expression cassette together with the FACS-based enrichment strategy presented here facilitates portability, thus serving as a blueprint for the development of tailored gene expression systems for a wide range of bacteria.

**IMPORTANCE** Reverse genetics is a powerful approach to study bacterial physiology and behavior by relying on well-developed genetic tools, such as inducible promoters. For the human pathogen Pseudomonas aeruginosa, well-characterized inducible promoters are scarce. In the current work, we used a synthetic biology-based approach to develop a cumate-inducible promoter for P. aeruginosa, termed P_QJ_, that shows excellent induction properties at the single-cell level. This genetic tool provides the means for qualitative and quantitative gene function studies describing P. aeruginosa’s physiology and virulence *in vitro* and *in vivo*. Because this synthetic approach to constructing species-specific inducible promoters is portable, it can serve as a blueprint for similar tailored gene expression systems in bacteria largely lacking such tools, including, for example, representatives of the human microbiota.

## INTRODUCTION

The opportunistic human pathogen Pseudomonas aeruginosa causes acute and chronic infections, posing a major and global human health risk (1–4). P. aeruginosa’s virulence mechanisms are widely studied, generally using model strains like PAO1 or PA14 (5, 6). Accordingly, the genetic toolbox for P. aeruginosa is well developed, including basic tools to efficiently delete genes, precisely integrate genes in defined chromosomal regions using transposons or integrases, and recently, to silence genes using CRISPR interference (7–13). However, well-characterized regulated gene expression systems are rather scarce, with many of the routinely used systems having been developed for other bacteria and adopted for use in P. aeruginosa without proper characterization or optimization (13, 14). In particular, since many of these systems predate modern single-cell analysis methods, it is often unclear how well they perform at the single-cell level in terms of population heterogeneity.

Many engineered gene expression systems rely on a promoter containing operator sites to which a transcriptional repressor binds in the absence of an inducer, thereby shutting down transcription (15). Binding of the inducer inactivates the repressor, resulting in derepression of the promoter and initiation of transcription. Ideally, regulated promoters specifically and uniquely respond to the intended inducer. However, many natural promoters show complex regulation, integrating multiple inputs or signals to tune gene expression. The use of heterologous expression systems is further complicated by the fact that they are often coupled to dedicated transport systems for the respective inducers (16–19) or have different RNA polymerase specificities for promoters (15).

An alternative approach to using naturally occurring regulated expression systems is the engineering of synthetic systems, where a minimal constitutively active core promoter (−35 and −10 boxes) of a specific organism is combined with operator sites and the corresponding repressor from a heterologous system (20–22). However, because it is difficult to predict the performance of combined promoter, operators, and repressor from different organisms, rational design of tunable promoters has remained challenging and generally requires cumbersome and time-consuming trial-and-error approaches (23).

In the current study, we set out to develop such a minimal synthetic inducible expression system for P. aeruginosa using semirandomized promoter libraries combined with the control elements from the heterologous 4-isopropylbenzoic acid (cumate)-inducible *cym*/*cmt* system (24, 25) and iterative rounds of fluorescence-activated cell sorting (FACS) as a powerful tool to enrich functional cumate-regulated promoter variants. The resulting promoter, termed P_QJ_, shows rapid induction kinetics with graded and homogeneous gene expression over a large dynamic range. P_QJ_ also offers expression that is strictly orthogonal to the widely used isopropyl β-d-thiogalactopyranoside (IPTG)-inducible *lacI*^q^-P_tac_ expression systems. Our work not only introduces a highly valuable genetic tool to study P. aeruginosa’s physiology and behavior but also offers a straightforward workflow for the development of inducible promoter systems in other bacteria.

## RESULTS

### Design of and screen for a cumate-inducible promoter for P. aeruginosa.

To develop a minimal synthetic inducible gene expression system for P. aeruginosa, we were inspired by a previously described cumate-inducible gene expression system engineered for *Alphaproteobacteria* (20). The original system relies on a strong synthetic σ^70^-dependent promoter specific for *Alphaproteobacteria*, P_syn2_, combined with two CuO operator sites up- and downstream from its −35 and −10 boxes, respectively, and the CuO-binding transcriptional repressor CymR from the heterologous cumate-inducible *cym*/*cmt* system originating from Pseudomonas putida strain F1. A high-GC codon-optimized version of *cymR* (denoted cymR*) was used in this system since *Alphaproteobacteria* are generally GC rich, and the expression of cymR* is driven by a modified *P_bla_* promoter, P_bla-mut1T_. The inducer cumate is inexpensive and membrane permeative, so it does not require dedicated transporters and can be readily used in both bacterial and eukaryotic cells. Moreover, P. aeruginosa does not have genes for cumate or cymene metabolism (24–27), insulating promoter control from intrinsic physiological changes. The original system developed for *Alphaproteobacteria* was designed in a modular manner, allowing easy exchange of core promoter sequences and, thus, offering a straightforward way to comprehensively screen core promoter libraries. Indeed, it has been shown before that the original core promoter can be readily exchanged with another alphaproteobacterial promoter, retaining inducibility. However, these studies were restricted to *Alphaproteobacteria* and the original system failed to respond to cumate in P. aeruginosa (Fig. S1 in the supplemental material), most likely because the original core promoter, P_syn2_, is inactive.

**FIG 1:**
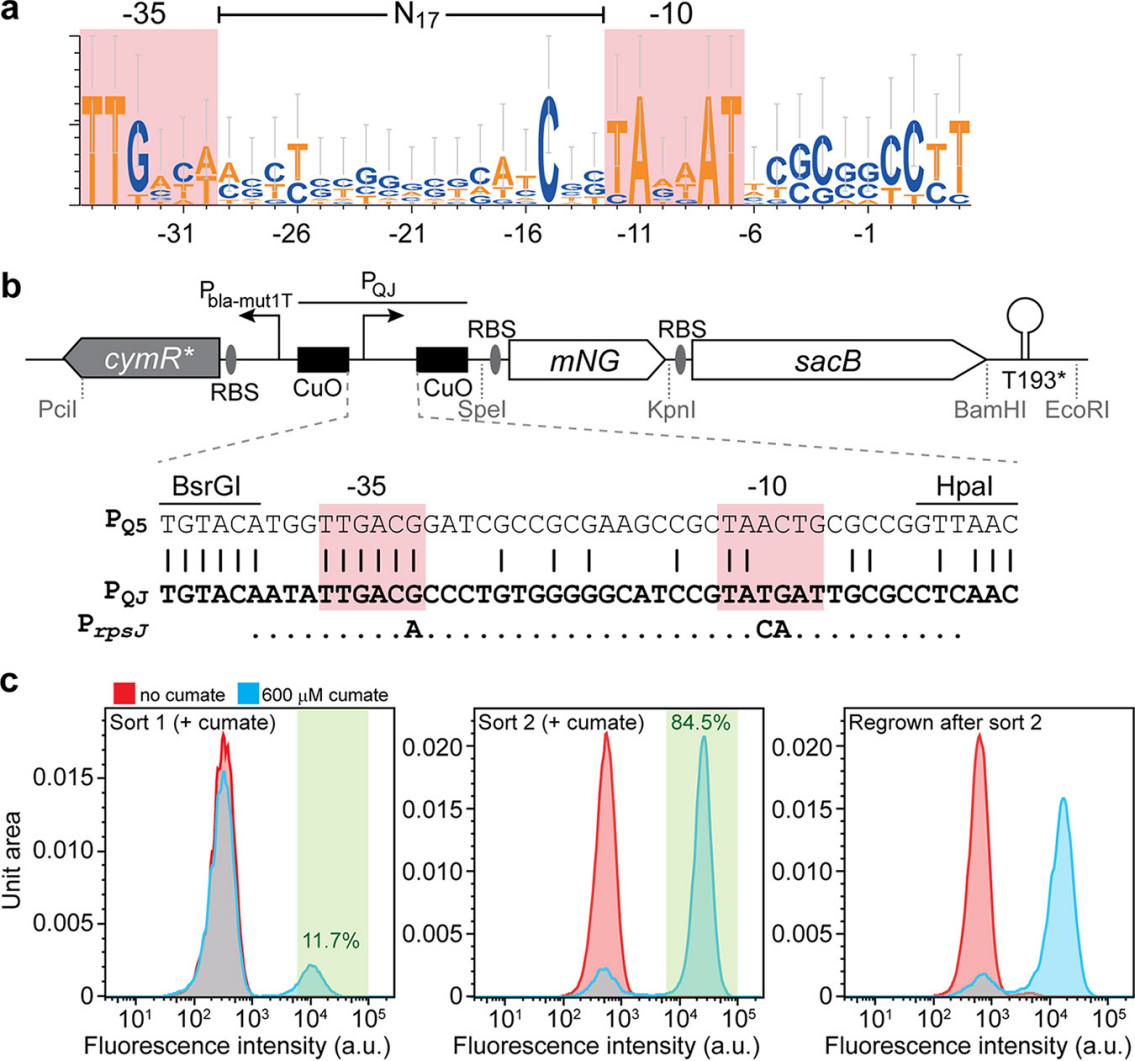
Isolation of cumate-inducible promoter P_QJ_. (a) Consensus sequence of an alignment of a set of putative P. aeruginosa PAO1 housekeeping gene promoters identified by a MEME motif search. −35 and −10 boxes are highlighted in red and labeled on top, and nucleotide positions relative to the predicted transcriptional start site (+1) are indicated at the bottom. Created using WebLogo 3. (b) The top shows a schematic of the cymR*-P_QJ_ regulatory region, including mNeonGreen (mNG), used as a fluorescence readout, and *sacB*, encoding levansucrase, which can potentially serve as a counterselection marker, found on the final plasmid isolated from the FACS screen. cymR* denotes a high-GC, codon-optimized version of *cymR*, P_bla-mut1T_ is a constitutively active mutant promoter derived from *P_bla_* driving cymR* expression, CuO sites are operator sites to which CymR binds, RBS indicates ribosomal binding sites, and T193* is a putative transcriptional terminator. The bottom shows an alignment of the original, alphaproteobacterial-specific promoter, P_Q5_, that was exchanged for P_QJ_ in the current study. BsrGI and HpaI restriction sites used to exchange P_Q5_ in *rpsJ* and *rpsT* promoter-based libraries are indicated above the sequences, as well as −35 and −10 boxes. The wild-type *rpsJ* promoter is included to highlight the changes in the final core promoter. (c) FACS profiles of cultures grown with and without cumate in naive libraries subjected to the first sort (left) and the second sort (middle) and after the second sort (right). The GFP^+^ gate applied in FACS is highlighted in green, and the percentages of cells in the cumate-induced samples that fell into this are given. a.u., arbitrary units.

To engineer a regulated synthetic promoter for P. aeruginosa, we first sought to identify the minimal core consensus of σ^70^-dependent promoters in P. aeruginosa strain PAO1 and therefore subjected regions upstream from putative housekeeping genes, mostly encoding small and large ribosomal subunit proteins (Data Set S1 in the supplemental material), to a motif search using MEME (28). The motif identified by this analysis, TTGACA-N_17_-TAAAAT (Fig. 1a; Data Set S2), is indicative of conserved −35 and −10 boxes properly spaced by 17 nucleotides and strongly resembles the σ^70^-dependent promoter consensus for E. coli, TTGACA-N_17_-TATAAT. In particular, the universally required A at position −11 is conserved in all predicted promoters. We chose the promoters of *rpsJ* (TTGACA-N_17_-TACAAT) and *rpsT* (TTGACA-N_17_-CATTAT) to replace the P_syn2_ promoter in the original system (Fig. 1b). Instead of using wild-type promoters, we generated libraries where nonconserved positions (−30, −12, −10, and −9) in the −35 and −10 boxes of the *rpsJ* and *rpsT* promoters were randomized. We used this approach because it has been shown previously that substitutions in these regions can substantially affect the strength and inducibility of engineered regulated gene expression systems (23). In these plasmid-borne, semirandomized libraries, promoters drive the expression of the fluorescent protein mNeonGreen (29) such that promoter activity with and without inducer can be inferred from fluorescence measurements. Libraries were transformed into P. aeruginosa PAO1, and cells were grown in the presence or absence of cumate. Flow cytometry indicated that a subset of cells responded to the addition of cumate and, thus, likely encoded functional, cumate-inducible promoters (Fig. 1c). We next isolated this highly fluorescent subpopulation by fluorescence-activated cell sorting (FACS). After regrowth with or without cumate, the fraction of cells responding to the addition of cumate was strongly enriched, and further sorting did not increase this fraction substantially. Isolation of individual clones from this population and subsequent sequencing of their promoter regions revealed a single promoter variant derived from *rpsJ* with the core promoter motif TTGACG-N_17_-TATGAT (Fig. 1b). In the following, we refer to this promoter variant as P_QJ_. We speculate that substitutions in the core promoter region reduce overall promoter strength and, at the same time, grant heterologous control by cumate. To confirm that the activity of P_QJ_ was regulated by cumate, we assayed the β-galactosidase activity of a P_QJ_-*lacZ* transcriptional fusion and verified that it also responded to cumate (Fig. S2a).

### P_QJ_ induction is rapid, graded, and highly homogenous.

**FIG 2:**
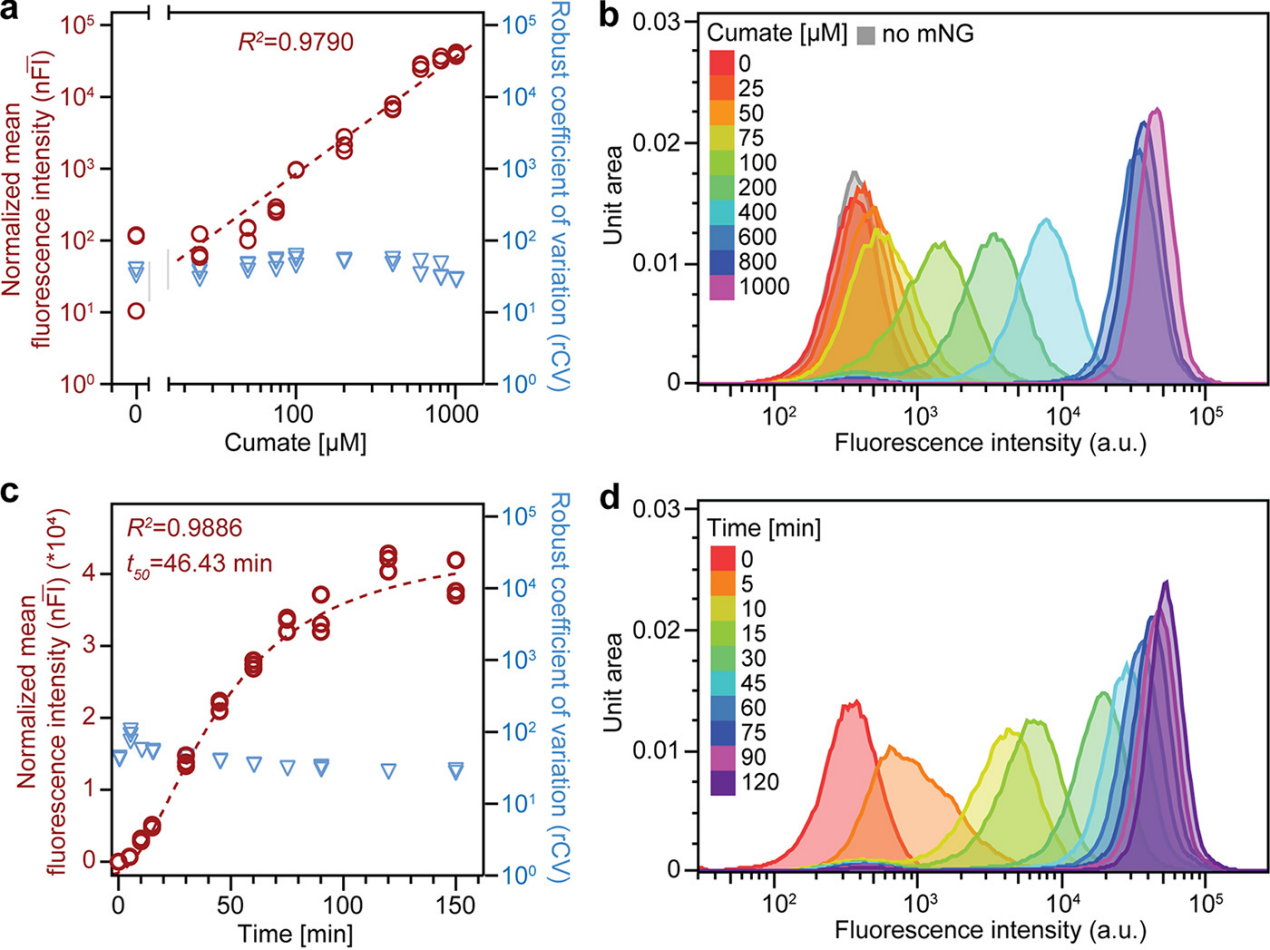
Dose-response curve and induction kinetics of P_QJ_ at the single-cell level. (a) Dose-response curve of strain UJP505 carrying plasmid pQFT-mNG. Symbols indicate individual mean values (left *y* axis) and robust coefficients of variation (rCV) (right *y* axis) from flow cytometry profiles from biological replicates (*n* = 3). The linear fit was performed using GraphPad Prism software on log-log-transformed values and excluded the samples with no cumate added. (b) Flow cytometry profile of one of the three biological replicates whose results are shown in panel a. Note that a UJP505 culture carrying a plasmid lacking mNeonGreen (pQFT) was included as a reference. (c) Induction kinetics of strain UJP505 carrying plasmid pQFT-mNG upon the addition of 1 mM cumate. Symbols indicate individual mean values from flow cytometry profiles from three biological replicates. The fit was performed using “[inhibitor] versus response − variable slope (four parameters) least-squares fit” (GraphPad Prism), and the goodness-of-fit *R^2^* value and the *t*_50_ value, describing the time it took to reach half-maximal induction, are shown. (d) Flow cytometry profile of one of the three biological replicates whose results are shown in panel c.

To quantitatively assess the performance of P_QJ_, we grew P. aeruginosa PAO1 carrying a plasmid expressing mNeonGreen under the control of P_QJ_ (pQFT-mNG) to exponential phase and performed dose-response curves with cumate concentrations ranging from 0 to 1 mM. Cumate at these concentrations had no effect on bacterial growth (Fig. S2b). As shown by the results in Fig. 2a, P_QJ_ activity showed a graded response to increasing cumate concentrations rather than switch-like behavior, suggesting that it allows fine-tuned expression of target genes. Without cumate, the mNeonGreen signal was indistinguishable from the signal in a strain carrying a control plasmid without mNeonGreen, suggesting that, in this experimental setup, P_QJ_ was tight, i.e., showed low leakiness in the absence of inducer. Induction was observed starting at a concentration of 50 μM cumate, and the log-log-transformed input-output function showed linearity between concentrations of 50 and 600 μM cumate. Between 600 μM and 1 mM cumate, the dose-response curve flattened, suggesting that the system reached saturation. Of note, a concentration of 1 mM cumate is close to the limit of solubility in aqueous solutions, for which reason we refrained from testing cumate concentrations higher than 1 mM. The flow cytometry profiles (Fig. 2b) showed unimodal distributions for all inducer concentrations tested, arguing that P_QJ_-dependent expression was highly homogenous across the population. These results demonstrate that P_QJ_ confers graded gene expression at the single-cell level, as opposed to an all-or-nothing behavior observed for other inducible promoters, such as the *araC-P_BAD_* or *lacI-P_lac_* systems of E. coli (16–19, 30–32). To more quantitatively assess homogeneity at the single-cell level, we also calculated the robust coefficient of variation (rCV), a measure of gene expression noise, for all concentrations of cumate tested (Fig. 2a). The rCV values remained rather constant at approximately 30 to 55 across all conditions, suggesting that all inducer concentrations resulted in comparable and narrow population distributions of gene expression.

**FIG 3:**
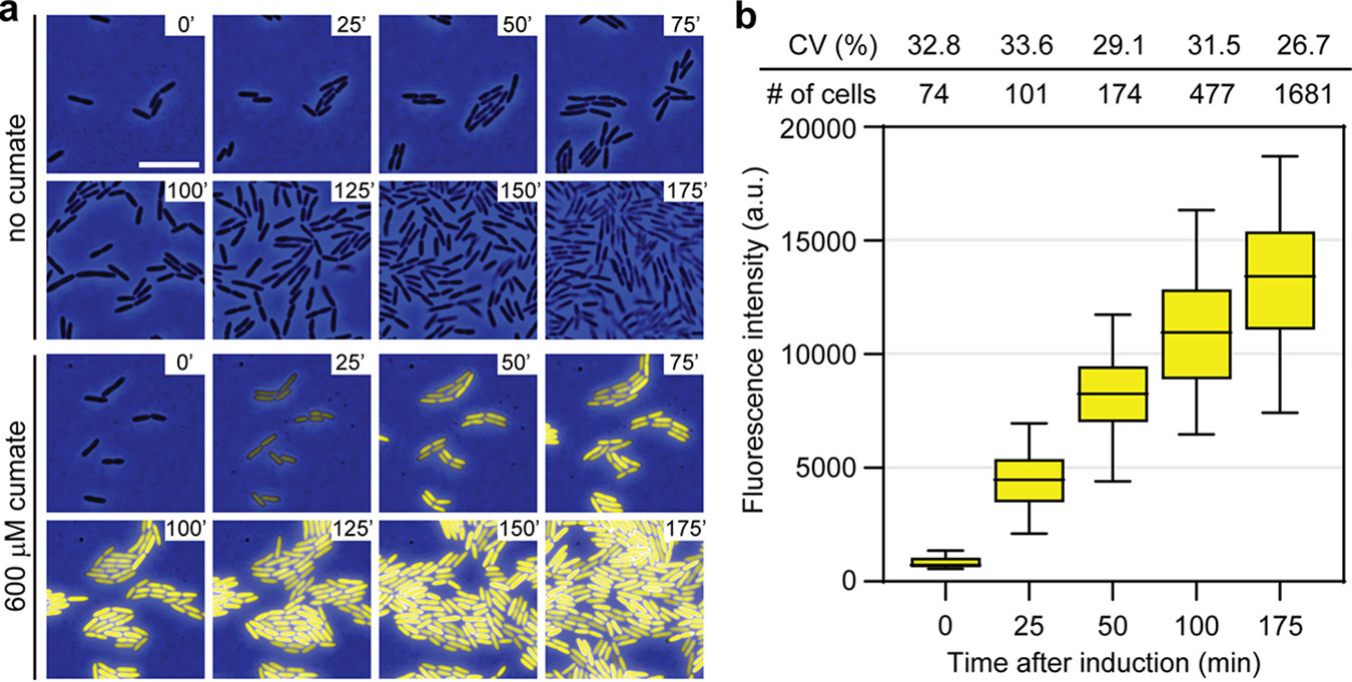
Induction kinetics of P_QJ_ in surface-associated cells. (a) Time-lapse microscopy of strain UJP505 harboring plasmid pQFT-mNG spotted on LB-Lennox 1% agarose pads with or without cumate at *t*_0_. Shown are false-colored composite images of the phase contrast channel (blue) and the green fluorescence channel (yellow). See Fig. S3a for individual channels. Scale bar = 10 μm. (b) Quantification of time-lapse data from the three biological replicates of the experiment whose results are shown in panel a. Boxes range from the lower to the upper quartile with the middle horizontal line representing the median; whiskers define the 5% and 95% percentiles. The coefficients of variation (CVs) and numbers of cells analyzed are given on top.

Next, we investigated the induction kinetics of P_QJ_. To this end, cells were grown to early exponential phase and induced with cumate at a concentration of 1 mM. Samples were taken at regular intervals after induction and analyzed by flow cytometry. The data presented in Fig. 2c and d show that a fluorescence signal was readily observed 5 min after the addition of the inducer. The fluorescence increased in a sigmoidal manner for approximately 120 min before reaching a plateau, with a fitted *t*_50_ (time to reach half-maximal induction) of around 46 min (Fig. 2c). These results suggested that P_QJ_ activity was immediately induced upon the addition of cumate and that inducer diffusion into the cell likely did not represent a major kinetic barrier for P_QJ_ induction. Inspection of the flow cytometry profiles (Fig. 2d) and calculation of the rCV (Fig. 2c) for all time points suggested that induction kinetics were homogenous across the population at the single-cell level. In order to evaluate these observations by an independent method, we turned to live-cell fluorescence microscopy. Exponentially growing cultures without cumate were spotted on LB agarose pads containing cumate (600 μM) and imaged every 5 min for several hours (Fig. 3a and b; Fig. S3; Movie S1). Fluorescence appeared as soon as 5 to 10 min after spotting cells on cumate-containing LB agarose pads, and the fluorescence signal increased over time and showed limited cell-to-cell variability, i.e., all descendants of the founding cells of developing microcolonies showed similar fluorescence intensities. No fluorescence was observed in control pads lacking cumate. Thus, these microscopy-based observations qualitatively confirmed the results from flow cytometry analyses.

We next tested the performance of P_QJ_ in P. aeruginosa PA14 (6), a more virulent relative of strain PAO1 within the genetically diverse P. aeruginosa group. Microscopy (Fig. S4a; Movie S2) and flow cytometry (Fig. S4b) indicated that strain PA14 suffered from frequent plasmid loss, for which reason we included a constitutively expressed reference red fluorescent protein, mScarlet-I, on the plasmid (pQFT-mNG-Pcon-Scar) (Fig. S4c) to gate for cells that maintained the reporter construct (Fig. S4d). Similar to the results for strain PAO1, P_QJ_ allowed graded and homogenous gene expression in PA14, although the dynamic range was reduced (Fig. S4e and f). Induction kinetics experiments revealed that a fluorescence signal was clearly observed 15 min after inducer addition and that the signal steadily increased over time in a mostly homogenous manner with a *t*_50_ of >75 min (Fig. S4g and h). While P_QJ_ is clearly functional in strain PA14 and shows induction properties similar to those in strain PAO1, the RK2-based plasmid replicon used here (33) seems to be especially instable in strain PA14 even under selection, such that it is advisable to transplant P_QJ_ on more stable replicons based on pBBR or pVS1 origins of replication in future studies.

Overall, these experiments establish P_QJ_ as a rapidly-responding, cumate-inducible promoter that allows homogenous and graded gene expression at the single-cell level, both in planktonic and surface-associated bacteria.

### P_QJ_-mediated gene expression is orthogonal to the *lacI*^q^-P_tac_ system.

**FIG 4:**
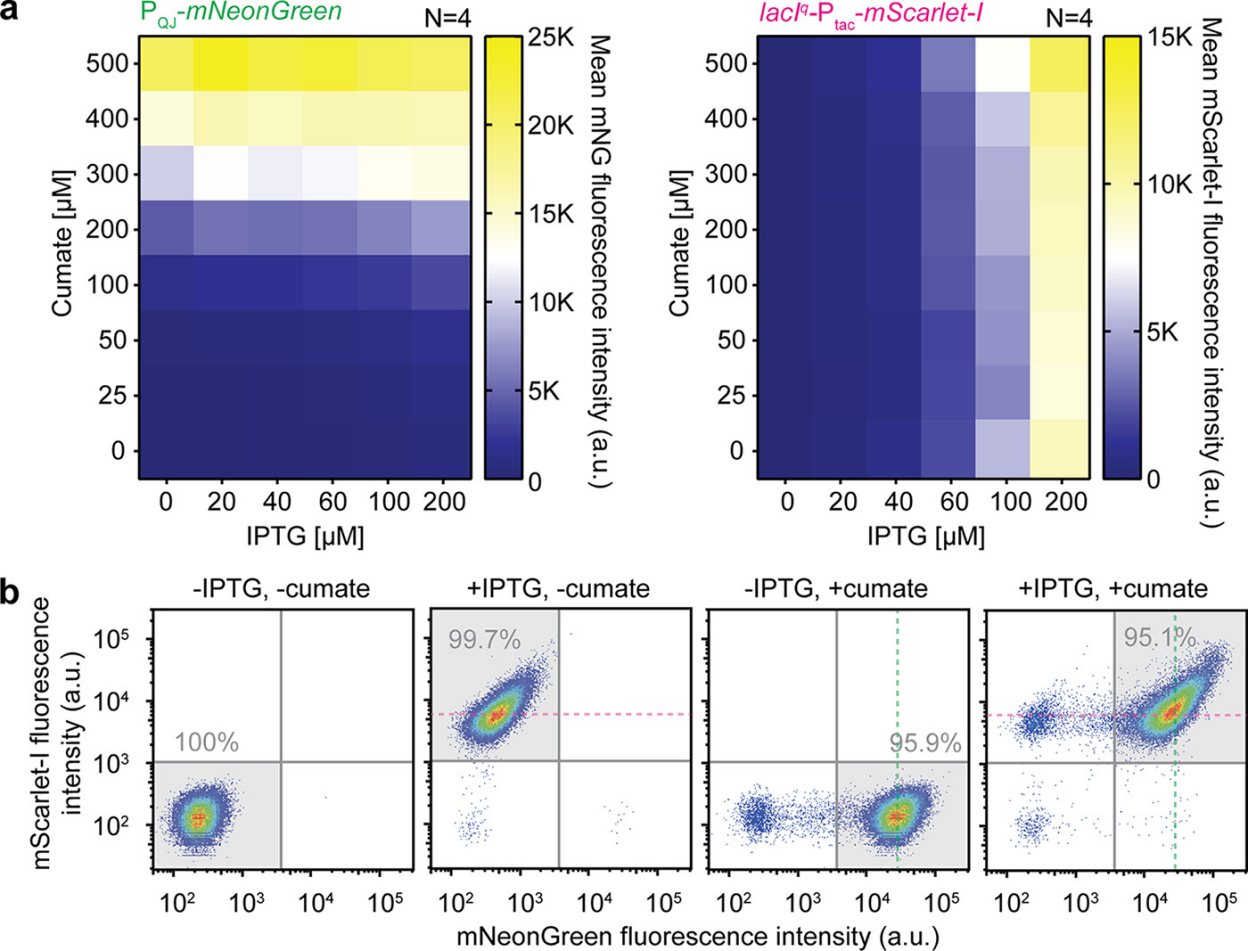
Orthogonality of P_QJ_ and *lacI*^q^-P_tac_. (a) Heat map representation of steady-state P_QJ_-mNeonGreen activity (left) and *lacI*^q^-P_tac_-mScarlet-I activity (right) of the same strain (UJP505 carrying both pQFTmNG and pGm6032-mScarlet-I) grown with different cumate and IPTG concentrations. Values represent the means from four biological replicates. The corresponding coefficients of variation are provided in Fig. S4. (b) Flow cytometry analysis of the same strain as in panel a grown with the indicated inducers. Four quadrants, indicating double-negative, single-positive, and double-positive cells, are shown and the percentages of cells in the highlighted quadrants (with gray background) are given. The green and magenta dotted lines indicate peak values of cultures grown in the presence of only cumate or only IPTG. Shown is one representative experiment from three biological replicates.

One of the most widely used inducible gene expression systems in P. aeruginosa is derived from the E. coli lac system, comprising the allolactose-inducible promoter of the *lacZYA* operon (in short, *P_lac_*) and its repressor LacI. In its natural context, *P_lac_* is also subject to regulation by glucose via the second messenger cAMP and the cAMP-binding transcriptional regulator CRP/CAP. Thus, in P. aeruginosa, a CRP/CAP-independent hybrid promoter called P_tac_ is usually used with a promoter-up *lacI* allele that shows increased expression levels called *lacI*^q^ and the nonhydrolyzable lactose analog IPTG as an inducer (34, 35). This so called *lacI*^q^-P_tac_ system is the basis for different expression platforms, such as pME6032- and miniTn*7*-based systems (7, 8, 36). In order to test whether P_QJ_ could be used in combination with the *lacI*^q^-P_tac_ system without regulatory interference, we constructed a strain expressing mNeonGreen under the control of P_QJ_ and the red fluorescent protein mScarlet-I (37) under the control of *lacI*^q^-P_tac_ from two separate plasmids. We deliberately chose this setup over expressing both reporters from a single plasmid or from the chromosome to be able to score the performance of P_QJ_ in combination with existing compatible plasmids. A P. aeruginosa strain harboring these two plasmids was grown in LB supplemented with different combinations of IPTG and cumate concentrations, and green and red fluorescence signals were recorded over time. As shown by the results in Fig. 4a, P_QJ_ and P_tac_ specifically responded to their designated inducers in a dose-dependent manner (also see Fig. S5). The addition of one inducer did not interfere with the response elicited by the other inducer, suggesting complete orthogonality of the two systems. To validate these results at the single-cell level, we grew the same strain to exponential phase in the presence or absence of IPTG (1 mM) and/or cumate (600 μM) and analyzed the cells using flow cytometry (Fig. 4b). The *lacI*^q^-P_tac_ system showed close to 100% induction at the single-cell level when treated with IPTG alone. The P_QJ_ system showed more than 95% induction-positive cells when treated with cumate alone. The addition of both cumate and IPTG resulted in >95% double-positive cells, essentially mimicking the combined responses to the individual inducers and, thus, confirming that the two systems were fully orthogonal at the single-cell level also.

## DISCUSSION

In this study, we developed a synthetic cumate-inducible gene expression system for P. aeruginosa called PQJ using the heterologous control elements from the P. putida strain F1 *cym*/*cmt* system in combination with a minimal constitutive promoter variant derived from a putative P. aeruginosa σ^70^-dependent housekeeping promoter. Using flow cytometry and live-cell microscopy, we characterized P_QJ_ in depth and showed that it responded rapidly and homogenously to the inducer cumate at the single-cell level, allowing graded gene expression. We also showed that P_QJ_ was fully orthogonal with the widely used semi-synthetic IPTG-inducible *lacI*^q^-P_tac_ system, enabling the integration of the P_QJ_-based expression system into many already-existing strains and genetic setups.

Compared to naturally occurring heterologous inducible gene expression systems, our synthetic approach circumvents a number of possible pitfalls. For example, the *araC-P_BAD_* system derived from E. coli is extremely leaky in P. aeruginosa due to the absence of glucose-mediated catabolite repression in this organism (14). Glucose-mediated catabolite repression in E. coli is mediated by the cAMP-binding transcription factor CRP/CAP, which directly binds to the *araC*-*P_BAD_* regulatory region (38, 39). Similarly, the wild-type *lacI-P_lac_* system from E. coli is subject to activation by cAMP-CRP/CAP in addition to repression by LacI (40, 41). Accordingly, one of the most frequently used inducible expression systems in P. aeruginosa, the *lacI*^q^*-P_tac_* system, employs a modified *P_lac_* promoter that lacks CRP/CAP operator sites and instead harbors a strong −35 box (34, 35). Complicating the situation even more, P. aeruginosa encodes a CRP/CAP homolog, Vfr, that can recognize the same operator sites as CRP/CAP, such that heterologous E. coli promoters containing these operator sites inadvertently respond to intrinsic physiological cues when used in P. aeruginosa (42–46). For example, the native CRP/CAP operator site-containing *P_lac_* promoter is used in P. aeruginosa as a sensor for cAMP, because cAMP-bound Vfr can act as a transcriptional activator of *P_lac_* (47). As an alternative, inducible systems naturally occurring in Pseudomonas can be used (e.g., see reference 48), but such systems come with their own downsides, e.g., instability of inducer concentration due to consumption, the physiological changes imposed upon inducer addition, bimodality due to feedback-regulated expression of dedicated transporters, and again, possible additional (unknown) regulatory inputs. Although some heterologous systems seem to function well in P. aeruginosa (14), fully synthetic systems generally avoid the many possible complications associated with natural systems described above *a priori*.

Importantly, we show that our cumate-regulated expression system functions well not only in planktonic cells but also in surface-associated cells. This suggests that P_QJ_ can also be used to study surface sensing, adherence, and biofilm formation, all of which play major roles in P. aeruginosa virulence and pathogenicity in acute and/or chronic infections (49). In addition, it was shown that the inducer cumate not only penetrates bacterial membranes but also readily enters mammalian cells in a manner that does not affect their growth, i.e., is nontoxic (27). Because neither P. aeruginosa nor mammalian cells seem to be capable of utilizing or degrading cumate (24–26), the inducer concentration is expected to remain stable over extended periods of time. These properties should allow the study of P. aeruginosa virulence in cell culture, tissue models, and possibly even animals.

By developing and rigorously characterizing the P_QJ_ inducible promoter system, we not only provide a novel, powerful genetic tool to study P. aeruginosa physiology, we also offer a convenient and general workflow for the construction of species-specific inducible expression systems. Specifically, this includes the identification of σ^70^-dependent minimal core promoters based on universally conserved genes, the generation of semirandomized libraries, and a rapid and powerful selection strategy based on FACS to identify functional cumate-inducible promoters. We propose that this workflow will serve as a blueprint for the development of tailored minimal synthetic (cumate-) inducible promoters for yet other organisms.

## MATERIALS AND METHODS

### Media, chemicals, and growth conditions.

Escherichia coli strain DH5α or DH5α T1R cells were used for cloning and made chemically competent according to the method described by Inoue et al. (50). All Pseudomonas aeruginosa PAO1 strains are based on the wild-type strain UJP505 (51), and PA14 strains are based on UJP209, a laboratory stock prepared from UCBPP-PA14 obtained from the Ausubel laboratory (6). Strains were grown in lysogeny broth (LB)-Miller medium (5 g/L yeast extract, 10 g/L tryptone, 10 g/L NaCl) or LB-Lennox medium (5 g/L yeast extract, 10 g/L tryptone, 5 g/L NaCl) at 37°C with shaking. Agar plates contained 1.5% (wt/vol) agar. Antibiotics were used when appropriate at the following final concentrations: oxytetracycline at 12.5 μg/mL for E. coli and 100 μg/mL for P. aeruginosa and gentamicin at 20 μg/mL for E. coli and 30 μg/mL for P. aeruginosa. Isopropyl β-d-thiogalactopyranoside (IPTG) stocks were prepared in double-distilled water (ddH_2_O) at a concentration of 1 M. 4-Isopropylbenzoic acid (cumate) was purchased from Sigma-Aldrich (catalog number 268402), and stocks were prepared in 100% ethanol at a concentration of 1 M. Note that the addition of cumate from an ethanol-based stock solution at a final concentration of 1 mM, the highest concentration tested, resulted in the precipitation of solid material (indicated by turbidity) that took <30 s to dissolve. Strains, plasmids, and oligonucleotides are listed in Tables 1 and 2.

**TABLE 1:** Strains and plasmids used in this study

Strain or plasmid	Description	Source or reference	Laboratory stock identification no.
Strains			
P. aeruginosa PAO1	P. aeruginosa PAO1 wild-type laboratory strain UJP505	51	UJP505
P. aeruginosa PA14	P. aeruginosa PA14 wild-type laboratory strain UJP209	6	UJP209
E. coli DH5α	F^−^ ϕ80*dlacZΔ*M15 Δ(*lacZYA-argF*)*U169 deoR recA1 endA1 hsdR17*(r_K_^−^ m_K_^+^) *phoA supE44* λ^−^ *thi-1 gyrA96 relA1*	Laboratory stock	UJ2710
E. coli DH5α T1R	T1 phage-resistant derivative of DH5α due to *tonA*/*fhuA* phenotype	Invitrogen	UJ6704
Plasmids			
pQF	Shuttle vector for cumate-inducible expression from P_Q5_; Tc^r^ IncP *oriV* ColE1 *oriV* RP4 *oriT*	20	UJ9524
pQF-mNG	pQF with *mNeonGreen* under control of P_Q5_	This study	UJ11231
pQF-mNGsacB	pQF with *mNeonGreen* and *sacB* under control of P_Q5_	This study	UJ11232
pQFT	pQF derivative with promoter P_Q5_ exchanged for promoter P_QJ_	This study	UJ11233
pQFT-mNG	pQFT with *mNeonGreen* under control of P_QJ_	This study	UJ11234
pQFT-mNGsacB	pQFT with *mNeonGreen* and *sacB* under control of P_QJ_	This study	UJP8111
pQFT-lacZ	pQFT with *lacZ* under control of P_QJ_	This study	UJ11235
pQFT-mNG-Pcon-Scar	pQFT-mNG with constitutively expressed *mScarlet-I*	This study	UJ11286
miniCTX1-mScarlet-I	mini-CTX1 with *mScarlet-I*	This study	UJP4763
mini-CTX1	Self-proficient integration vector for integration at the chromosomal ϕCTX *att* site in P. aeruginosa; Tc^r^	10	UJP4092
pGm6032	Gm-resistant derivative of pME6032, described as an intermediate plasmid in the construction of pGm-*yfiprom-N* in reference 55; Gm^r^ pVS1 *oriV* p15A *oriV*	55	UJP1142
pGm6032::mScarlet-I	pGm6032 with *mScarlet-I* under control of *lacI*^q^-P_tac_	This study	UJP6833

**TABLE 2:** Oligonucleotides used in this study

Oligonucleotide	Sequence (5′ to 3′)
14153	GTACAATATTGACNCCCTGTGGGGGCATCCGTANNATTGCGCCTC
14154	GAGGCGCAATNNTACGGATGCCCCCACAGGGNGTCAATATT
14155	GTACCCCCTTGACNACCCCTGGAGGCGACGGCANNATTCGCGGCC
14156	GGCCGCGAATNNTGCCGTCGCCTCCAGGGGTNGTCAAGGGG
14157	ATTTTGGTACCTTATTTATACAGCTCATCCATACCC
14158	ATTTTACTAGTAGAGGAAGCTTCCGCATGGTGAGCAAAGGTGAAGAGGATAAT
14647	GTACCCCCNNNACAACCCCTGGAGGCGACGGCAATATTCGCGGCC
14648	GGCCGCGAATATTGCCGTCGCCTCCAGGGGTTGTNNNGGGG
14649	GTACCCCCTTGNNNACCCCTGGAGGCGACGGCAATATTCGCGGCC
14650	GGCCGCGAATATTGCCGTCGCCTCCAGGGGTNNNCAAGGGG
14651	GGAAAAACGTATCAAAACGTACAGCAGTTCATCGATG
14652	AAACACTGACAACTGCACAAGTTAATGTATCAGCATCAGACAGC
14653	TGGCCTTTTTTTTTGCGGGTCACTTCACCGGATCCTTATTTATTAACTGTTAATTGTCCTTG
14654	GGGTATGGATGAGCTGTATAAATAAGGTACCAAATGAACATCAAAAAGTTTGCAAAAC
14655	CATCGATGAACTGCTGTACGTTTTGATACGTTTTTCC
14656	GCTGTCTGATGCTGATACATTAACTTGTGCAGTTGTCAGTGTTT
14884	TAAACTCGAGAAGCAAACCCGTACGTAGGAGGAGTAATGAGCAAAGGTGAAGCCG
14885	ATTGGGTACCTTATTTATACAGTTCATCCATACCACC
19739	CGATGTGATGGGTATGGATGAGCTGTATAAATAAGGTACCTTTATAGCTAGCTTGACATCCCATAGAGTGATAGAG
19740	TTGACATCCCATAGAGTGATAGAGATACTGAGCACAaagaaaCACGAGGAAAACTAAACTAGTATGAGCAAGAAATATGG
19741	GCCGTCGTGGTCCTTGTAGTCCGGATCCCAATTGGAGCTCTTTTTTACTAGCTCACTTGTACAGTTCATCCATACC

### Plasmid delivery into P. aeruginosa by electroporation.

P. aeruginosa PAO1 or PA14 cells were inoculated from single colonies or cryostocks and grown overnight in glass tubes as 5-mL cultures in LB-no salt (5 g/L yeast extract, 10 g/L tryptone) at 37°C with shaking (180 rpm). To prepare cells for electroporation, 800 μL of an ON culture was spun down in a tabletop microcentrifuge for 30 s at maximum speed. The supernatant was discarded, and the pellet was washed 2 to 3 times with 800 μL of ddH_2_O. The final pellet was dissolved in an appropriate volume of ddH_2_O, typically 100 to 500 μL. One-microliter amounts of plasmid were added to 100 μL of cells in 2-mm cuvettes, and electroporation was performed using a Bio-Rad GenePulser with the following settings: 25 μF, 400 Ω, 2.5 kV. All steps described were performed at room temperature. Amounts of 500 to 800 μL of LB-Miller were added immediately after the pulse, and cells were incubated for 1 to 2 h at 37°C with shaking before plating on selective plates.

### *In silico* analysis of housekeeping gene promoters.

Upstream regions of putative P. aeruginosa PAO1 housekeeping genes, mostly coding for ribosomal proteins, were extracted from the reference genome sequence (accession number NC_002516) (26) and subjected to a motif search using MEME (28) with the following specifications: distribution of motif occurrences, 0 or 1 per sequence; number of different motifs, 15; minimum number of sites, 5; maximum number of sites, 30; minimum motif width, 20; maximum motif width, 50; searching, “given strand only.” Based on these results, a consensus promoter sequence motif was generated using WebLogo 3 (52).

### Plasmid construction.

pQF-mNG was constructed by PCR amplification of a fragment containing mNeonGreen (mNG; the template was a gift from Dirk Bumann, Biozentrum, University of Basel) with primer pair 14157/14158, digestion with KpnI/SpeI, and cloning in the same sites of pQF (20). Note that primer 14157 introduced a silent point mutation that eliminated a BsrGI restriction site close to the end of the mNG coding sequence. For construction of pQF-mNGsacB, three silent mutations in *sacB* were introduced to eliminate internal BsrGI and HpaI restriction sites by PCR amplification of three *sacB* fragments using primer pairs 14654/14656, 14652/14655, and 14651/14653 and pNPTStet (53) as a template for *sacB*. The three fragments were joined by splicing by overhang extension (SOE) PCR using primer pair 14653/14654. The resulting PCR product was then cloned in pQF-mNG digested with KpnI/BamHI via Gibson assembly (54). The original plasmid pQFT-mNGsacB resulted from the FACS screen for cumate-regulated promoters described below, and a PciI/SpeI fragment from this plasmid was subcloned in pQF-mNGsacB to obtain a clean version of pQFT-mNGsacB that was used in all following steps. Plasmid pQFT-mNG was obtained by subcloning an SpeI/EcoRI fragment encoding mNG from pQF-mNG into pQFT-mNGsacB digested with the same restriction enzymes. Plasmid pQFT-*lacZ* was obtained by subcloning an XbaI/EcoRI fragment encoding *lacZ* from pAK127lacZ(MCS) (20) in pQFT-mNGsacB digested with SpeI/EcoRI. Plasmid pQFT was constructed by subcloning a PciI/SpeI fragment carrying cymR* and the modified promoter from pQFT-mNG in pQF digested with the same enzymes. miniCTX1-mScarlet-I was constructed by PCR amplification of a fragment containing mScarlet-I (the template was a gift from Marek Basler, Biozentrum, University of Basel) with primer pair 14884/14885, digestion with KpnI/XhoI, and cloning in the same sites of mini-CTX1 (10). pGm6032::mScarlet-I was constructed by subcloning a KpnI/EcoRI fragment carrying mScarlet-I from miniCTX1-mScarlet-I in pGm6032 (55) digested with the same enzymes. For construction of plasmid pQFT-mNG-Pcon-Scar, a fragment encoding mScarlet-I was amplified from pConRef-2H12 (56) by PCR in a reaction mixture containing primers 19739 (400 nM), 19749 (13 nM), and 19741 (400 nM) and cloned in pQFT-mNG digested with KpnI/SacI via Gibson assembly. Oligonucleotides 19739 and 19740 introduced a putative constitutively active promoter upstream from mScarlet-I, the design of which was based on the PLtetO-1 promoter (57). To render PLtetO-1 independent of TetR control, this promoter lacked the TetO operator sequence upstream from the −35 box and had nucleotides changed in the TetO sequence interspersing the −35 and −10 boxes in PLtetO-1. After transformation of the Gibson reaction into E. coli DH5α, a plasmid from a bright red colony was isolated and sequenced. The isolated clone had an additional base in the −10/−35 spacer compared to the intended design, which made the spacing of the original −35 and −10 boxes suboptimal (18-nucleotide [nt] linker instead of 17 nt) but simultaneously generated a second putative active promoter (TTTATA-N_17_-TAGAAT). We refer to this putative dual hybrid promoter as P_hyb18_ (see Fig. S3c) and used it for follow-up experiments since it also gave a robust red fluorescence signal in P. aeruginosa. Plasmids will be made available through Addgene.

### Promoter library construction.

Double-stranded DNA (dsDNA) fragments for P. aeruginosa promoter libraries were generated by separately phosphorylating and annealing oligonucleotide pairs 14153/14154, 14155/14156, 14647/14648, and 14649/14650 following a previously published protocol (12) with the following modifications: primers were annealed in a thermocycler using consecutive 3-min incubations at 95°C, 90°C, 80°C, 70°C, 60°C, 50°C, 40°C, 30°C, and 20°C, and annealed oligonucleotides were stored undiluted at −20°C until further use. Libraries were constructed by incubating 200 ng of BsrGI/HpaI-digested pQF-mNGsacB with promoter library dsDNA (annealed oligonucleotides 14153/14154, 14155/14156, 14647/14648, and 14649/14650; 167 nM each) and 400 units of T4 DNA ligase (NEB) in 15 μL 1× T4 DNA ligase buffer (NEB) for 1 h at room temperature, transformation of 10 μL of the ligation mixture into in-house-prepared chemocompetent E. coli DH5α, and plating on LB supplemented with oxytetracycline. Approximately 5,000 colonies were obtained, which covers the number of theoretically possible promoter variants (4 × 4^3^ = 256) by approximately 20-fold. All colonies were pooled, and plasmid DNA was extracted using a GenElute plasmid DNA miniprep kit (Sigma) to obtain the promoter library.

### FACS: isolation of pQFT.

The promoter library was transformed into P. aeruginosa PAO1 strain UJP505 by electroporation, and cells were plated on LB agar plates containing oxytetracycline and sucrose (8% [wt/vol]) following phenotypic expression for 2 h at 37°C. After incubation for 24 h at 37°C, colonies were pooled in 1 mL of LB-Miller. This suspension was diluted 1,000-fold to start two 50-mL LB cultures containing oxytetracycline—one supplemented with 600 μM cumate and one mock treated with ethanol. After 4 h of growth at 37°C, aliquots from cultures were taken and diluted in phosphate-buffered saline (PBS), and mNG fluorescence was measured using a 488-nm blue laser, a 495-nm long-pass (LP) mirror, and a 514-/30-nm band-pass (BP) filter on a BD Aria III cell sorter (FACS core facility, Biozentrum, University of Basel). Cells (ca. 50,000) from the culture grown with cumate that showed high mNG fluorescence signals were sorted in 5 mL of LB supplemented with oxytetracycline. After regrowth of these cells overnight, 20 μL of the culture was used to inoculate two 5-mL LB-Miller cultures containing oxytetracycline, one supplemented with 600 μM cumate and one mock treated with ethanol, and the cultures were grown and measured by flow cytometry as described above. Cells (ca. 50,000) from the culture grown with cumate were sorted for high fluorescence and collected as before. After overnight incubation at 37°C with shaking, plasmid DNA was extracted using a GenElute plasmid DNA miniprep kit (Sigma) and retransformed into chemocompetent E. coli DH5α. Plasmid DNA was extracted from this E. coli pool and sent for sequencing with primer 10572 (Microsynth, Balgach, Switzerland) to identify promoter variants. The sequencing result was unambiguous with a clean chromatogram, suggesting that a single promoter variant dominated the pool. Retransformation of this pool into E. coli DH5α and isolation of plasmids from individual clones followed by sequencing revealed identical sequences for all clones tested, matching the pool sequencing results. The promoter variant derived from the *rpsJ* promoter, and we named the resulting promoter P_QJ_.

### Flow cytometry.

For dose-response measurement, an overnight culture of strain UJP505 or UJP209 carrying pQFT-mNG or pQFT-mNG-Pcon-Scar was grown in LB-Miller (UJP505) or LB-Lennox (UJP209) with oxytetracycline, diluted 1:100 in 5 mL of the same medium with different cumate concentrations, and grown for 3 to 4 h. Aliquots were taken and diluted 1:20 in phosphate-buffered saline (PBS) and then measured on a BD LSR Fortessa II using Diva software (BD Biosciences). For induction kinetics experiments, an overnight culture was grown in LB-Miller (UJP505) or LB-Lennox (UJP209) with oxytetracycline, diluted 1:100 in 5 mL of the same medium, and grown until reaching an optical density at 600 nm (OD_600_) of 0.3 to 0.4. Then, an aliquot was taken from the culture (*t*_0_) and diluted 1:20 in PBS containing 30 μg/mL chloramphenicol (to inhibit translation), and 1 mM cumate was added to the remaining culture to induce P_QJ_. At indicated time points, the culture was sampled by taking aliquots and diluting them 1:20 (or 1:50 with increasing density) in PBS containing 30 μg/mL chloramphenicol. All samples were measured in parallel on a BD LSR Fortessa II using Diva software (BD Biosciences). For orthogonality experiments, stationary overnight cultures of strain UJP505 carrying pQFT-mNG and pGm6032::mScarlet-I in LB-Lennox containing oxytetracycline and gentamicin were diluted 1:100 in 5 mL of the same medium and grown for another 3 h. Then, cumate and/or IPTG was added, followed by incubation for another 2 to 3 h before sampling in PBS and analysis on a BD LSR Fortessa II using Diva software (BD Biosciences). For flow cytometry analysis, following standard side scatter height (SSC-H)/forward scatter height (FSC-H) and SSC-H/side scatter width (SSC-W) gating for singlets, events were analyzed for mScarlet-I using a yellow-green 561-nm laser, a 600-nm long-pass mirror, and a 610-/20-nm band-pass filter and for mNeonGreen using a blue 488-nm laser, a 505-nm long-pass mirror, and a 512-/25-nm band-pass filter. The gating strategy used for UJP209 carrying plasmid pQFT-mNG-Pcon-Scar is outlined in Fig. S4c. Data were analyzed using FlowJo version 10.0.6 (FlowJo LL), R, and GraphPad Prism 8 or Prism 9. For quantification of fluorescence in the experiments whose results are shown in Fig. 2a and c, a strain carrying a control plasmid without mNeonGreen (UJP505 pQFT) was included and used for background correction. The robust coefficient of variation (rCV) values were taken from the in-built statistical analysis provided by FlowJo.

### Microscopy.

Cultures of strain UJP505 or UJP209 carrying pQFT-mNG were grown overnight in LB-Lennox supplemented with oxytetracycline at 37°C, diluted 1:1,000 in the same medium, and grown for another 3 h at 37°. Amounts of 2 μL of cultures were spotted on LB-Lennox 1% agarose pads containing oxytetracycline with or without 600 μM cumate, and images were acquired every 5 min using a DeltaVision system with softWoRx 6.0 (GE Healthcare) on an Olympus IX71 microscope equipped with a pco.edge scientific complementary metal oxide semiconductor (sCMOS) camera and an UPlan FL N 100×/1.30 oil objective (Olympus) at 37°C. The mNeonGreen signal was recorded using standard green fluorescent protein (GFP) excitation and emission filters with 100-ms exposure time at 50% power. The ImageJ plugin MicrobeJ (58) was used for quantification of single-cell fluorescence signals on time-lapse data from three biological replicates with two fields of view each.

### β-Galactosidase assays.

β-Galactosidase assays were performed as previously described (59). In short, overnight cultures of strain UJP505 carrying pQFT-*lacZ* grown in LB-Miller containing oxytetracycline were diluted 1:100 in the same medium supplemented with the indicated cumate concentration. After 3.5 h, the OD_600_ was measured and 20 μL of each culture was used and mixed with a premeasured 80-μL amount of permeabilization solution in 1.5-mL microfuge tubes. The samples were incubated at 30°C for at least 30 min before the addition of 600 μL prewarmed substrate solution. After sufficient color had developed, 700 μL of stop solution was added and the total time of the enzymatic reaction was taken. The 1.5-mL microfuge tubes were then spun down for 5 to 10 min at full speed, and the supernatant was transferred into cuvettes, allowing measurement at OD_420_ and calculation of Miller units.

### Plate reader measurements.

Orthogonal expression and growth curve experiments were performed using a Synergy H1 hybrid multimode reader (BioTek Instruments). For growth curve experiments, overnight bacterial cultures were grown in LB-Miller supplemented with oxytetracycline and washed once before adjusting the OD_600_ to 1 in the same medium. These were then used to inoculate the wells of a clear flat-bottom 96-well plate to an OD_600_ of 0.005, and the OD_600_ was read every 10 min over 24 h with the following settings: preheating to 37°C and double-orbital continuous shaking with a frequency of 425 cpm (3 mm). For orthogonal expression experiments, an overnight culture of UJP505 carrying pQFT-mNG and pGm6032::mScarlet-I grown in LB-Lennox containing oxytetracycline and gentamicin was diluted 1:1,000 in 5 mL of the same medium and grown for 4 h. Then, this culture was diluted 10-fold in LB-Lennox and 2-μL amounts of this dilution were used to inoculate wells containing 180 μL of LB with appropriate antibiotics and cumate and IPTG concentrations as indicated. The plate reader settings were the same as described above for growth curve experiments, except that green and red fluorescence was measured in addition using the following settings: excitation, 500 nm; emission, 530 nm; bottom, gain of 80; and excitation, 565 nm; emission, 595 nm; bottom, gain of 80. For analysis, first the OD_600_ and fluorescence values were background corrected by subtracting the average values of wells containing medium only. Then, fluorescence signals were normalized to the number of cells by dividing, for each time point, the red and green fluorescence signal values by the corresponding OD_600_ value. Finally, the fluorescence values for each growth curve were averaged from all time points where the OD_600_ was between 0.2 and 0.6, ensuring that the readouts from all conditions were comparable. This resulted in a single mNeonGreen and mScarlet-I value for each condition, and the mean values from four independent biological replicates are shown in the final heat maps. Figure S5 shows the corresponding coefficients of variation, calculated by dividing the standard deviations by the mean values.

### Data availability.

The data that support the findings of this study are available from the corresponding authors upon reasonable request.
