# Supplementary material for: A Synthetic Cumate-Inducible Promoter for Graded and Homogenous Gene Expression in Pseudomonas aeruginosa
Source: Appl Environ Microbiol. 2023 May 18;89(6):e00211-23. doi: 10.1128/aem.00211-23 (PMC10304978; doi:10.1128/aem.00211-23)
Supplement: Supplemental file 1 — Supplemental material. Download aem.00211-23-s0001.pdf, PDF file, 0.6 MB [file aem.00211-23-s0001.pdf]

SUPPLEMENTAL MATERIAL LEGENDS FOR

A synthetic cumate-inducible promoter for graded and homogenous gene expression in *Pseudomonas aeruginosa*

Alexander Klotz[1,2,3], Andreas Kaczmarczyk[1,2], Urs Jenal[2]

Biozentrum, University of Basel, Spitalstrasse 41, 4056 Basel, Switzerland

[1] Equal contribution

[2] Correspondence to: alexander.klotz@unibas.ch, andreas.kaczmarczyk@unibas.ch or urs.jenal@unibas.ch

[3] Current address: Department of Biosystems Science and Engineering (D-BSSE), Swiss Federal Institute of Technology Zürich (ETHZ), Mattenstrasse 26, 4058 Basel, Switzerland; alexander.klotz@bsse.ethz.ch

Supplementary Fig. 1 – P_{Q5} activity in *P. aeruginosa* PAO1

Flow cytometry profile of one of the three biological replica of strain UJP505 carrying plasmid pQF-mNG, encoding mNeonGreen under control of the original cumate inducible promoter, P_{Q5}, developed for Alphaproteobacteria. Note that a UJP505 culture carrying a plasmid lacking mNeonGreen (pQFT) was included as a reference point.

Supplementary Fig. 2 – P_{QJ}-*lacZ* activity and effect of cumate on growth

a. Activity of a P_{QJ}-*lacZ* transcriptional fusion in PAO1 (strain UJP505 carrying pQFT-lacZ) with different inducer concentrations. Shown are individual values from three biological replicates (orange circles) as well as mean and standard deviations.
b. Growth curves of indicated strains without or with indicated cumate concentrations. Shown are individual data points for three independent biological replicates.

Supplementary Fig. 3 – Time-lapse microscopy of P_{QJ} induction kinetics

a. Time-lapse microscopy of strain UJP505 harboring plasmid pQFT-mNG spotted on LB 1% agarose pads with or without cumate at t_0. Shown are individual phase contrast (Phase) and green fluorescence (mNG) channels of the same data shown in **Fig. 3a**. Scale bar: 10 μm.
b. Time-lapse microscopy of strain UJP505 harboring plasmid pQFT-mNG spotted on LB 1% agarose pads with cumate at t_0. Note that exactly the same data as in panel a and **Fig. 3a** are shown, but with higher temporal resolution and adjusted contrast to better visualize P_{QJ} induction at early time points. Shown are individual phase contrast (Phase) and green fluorescence (mNG) channels and false-colored composite images of the phase contrast channel (blue) and the green fluorescence channel (yellow). Scale bar: 10 μm.

Supplementary Fig. 4 – Characterization of P_{QJ} in *P. aeruginosa* PA14

a. Time-lapse microscopy of strain UJP209 harboring plasmid pQFT-mNG spotted on LB 1% agarose pads with cumate at t_0. Shown are individual phase contrast (Phase) and green fluorescence (mNG) channels and false-colored composite images of the phase contrast channel (blue) and the green fluorescence channel (yellow). Arrows point at cells that have lost plasmid pQFT-mNG as judged by lack of fluorescence 90 min after cumate addition. The cell highlighted by the magenta arrow does not divide at all, whereas the cells highlighted by the white arrow manage to undergo a few divisions before growth ceases.
b. Flow cytometry profiles of the dose-response curve of strain UJP505 carrying plasmid pQFT-mNG. Note that a UJP209 culture carrying a plasmid lacking mNeonGreen (pQFT-lacZ) was included as a reference.
c. Schematic of pQFT-mNG-Pcon-Scar expressing the red fluorescent protein mScarlet-I as a reference from the constitutively active promoter P_{hyb18}. Highlighted in red and in bold are the -35 and -10 boxes derived from the PLtetO-1 promoter. Alternative -35 and -10 boxes (see Materials

and Methods for details) are highlighted in grey and are in italics. The ribosome binding site and mScarlet-I start codon are in indicated in bold italic. All other elements are as described in Fig. 1b.

d. Gating strategy used for strain UJP209 carrying pQFT-mNG-Pcon-Scar to assay PQJ activity in cells that have retained the plasmid.
e. Flow cytometry profiles of the dose-response curve of strain UJP505 carrying plasmid pQFT-mNG-Pcon-Scar.
f. Dose-response curve of strain UJP209 carrying plasmid pQFT-mNG-Pcon-Scar. Symbols indicate individual median values (left axis) and robust coefficients of variation (rCV; right axis) from flow cytometry profiles – as shown in panel e – of biological replica (N=3). The linear fit was performed using GraphPad Prism software on log-log-transformed values and excluded the samples with no cumate added.
g. Flow cytometry profiles of induction kinetics of strain UJP209 carrying plasmid pQFT-mNG-Pcon-Scar upon addition of 1 mM cumate.
h. Dose-response curve of strain UJP209 carrying plasmid pQFT-mNG-Pcon-Scar. Symbols indicate individual median values (left axis) and robust coefficients of variation (rCV; right axis) from flow cytometry profiles – as shown in panel g – of biological replica (N=3). No fit was applied since fluorescence did not plateau over the course of the experiment.

Supplementary Fig. 5 – Coefficents of variations related to Fig. 4a

Heat map representation coefficients of variations (CVs) of steady-state P_{QJ}-mNeonGreen activity (left panel) and *lacI*^{q}-P_{tac}-mScarlet-I activity (right panel) of the same strain (UJP505 carrying both pQFTmNG and pGm6032-mScarlet-I) grown with different cumate and IPTG concentrations. Values represent are from four biological replicates and relate to **Fig. 4a**.

Supplementary Movie 1

Time-lapse of P_{QJ} induction kinetics of strain UJP505 carrying pQFT-mNG. At t_0 cells were spotted on LB-Lennox 1% agarose pads with or without cumate.

Supplementary Movie 2

Time-lapse of P_{QJ} induction kinetics of strain UJP209 carrying pQFT-mNG. At t_0 cells were spotted on LB-Lennox 1% agarose pads with or without cumate. Note that cells that do not show fluorescence cease growth and eventually stop dividing, indicating that they have lost the plasmid and thus the ability to grow in the presence of tetracycline.

Supplemental Data 1

Putative housekeeping gene promoter sequences used for MEME search (MEME input; in fasta format)

Supplemental Data 2

Alignment of putative housekeeping gene core promoters with the conserved motif identified by MEME (MEME output; in fasta format)
