# Supplementary figures and images for: A Synthetic Cumate-Inducible Promoter for Graded and Homogenous Gene Expression in Pseudomonas aeruginosa
Source: Appl Environ Microbiol. 2023 May 18;89(6):e00211-23. doi: 10.1128/aem.00211-23 (PMC10304978; doi:10.1128/aem.00211-23)

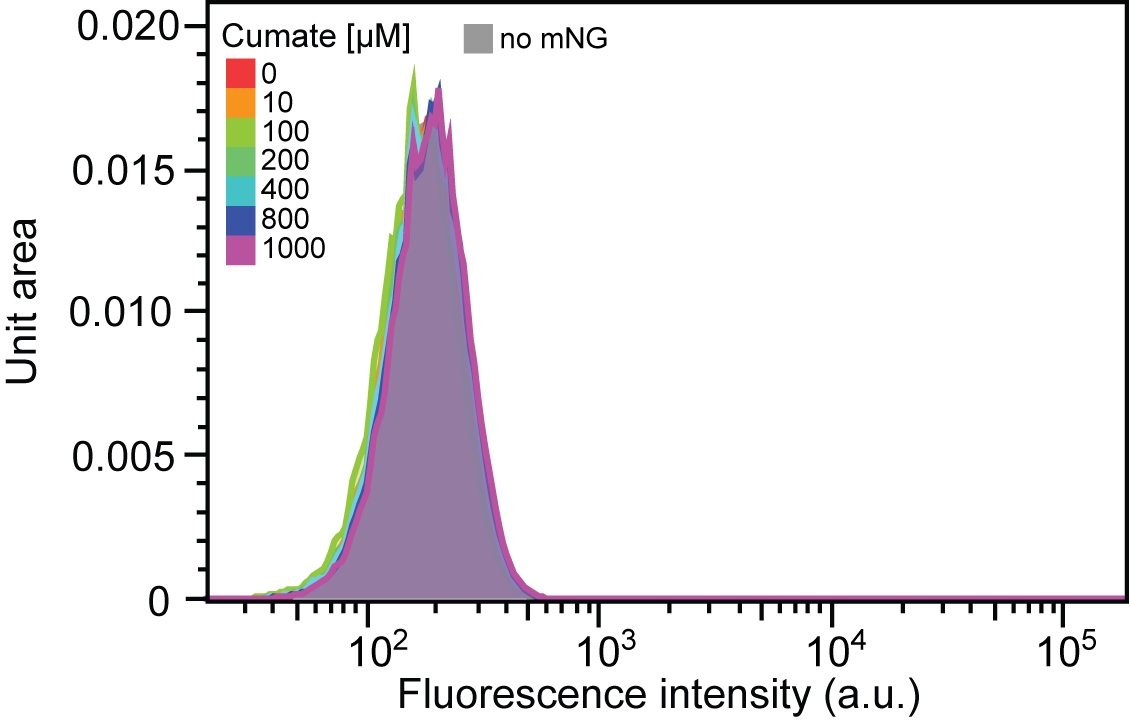

Supplement: Supplemental file 2 — Supplemental material. Download aem.00211-23-s0002.tif, TIF file, 0.3 MB [file aem.00211-23-s0002.tif]

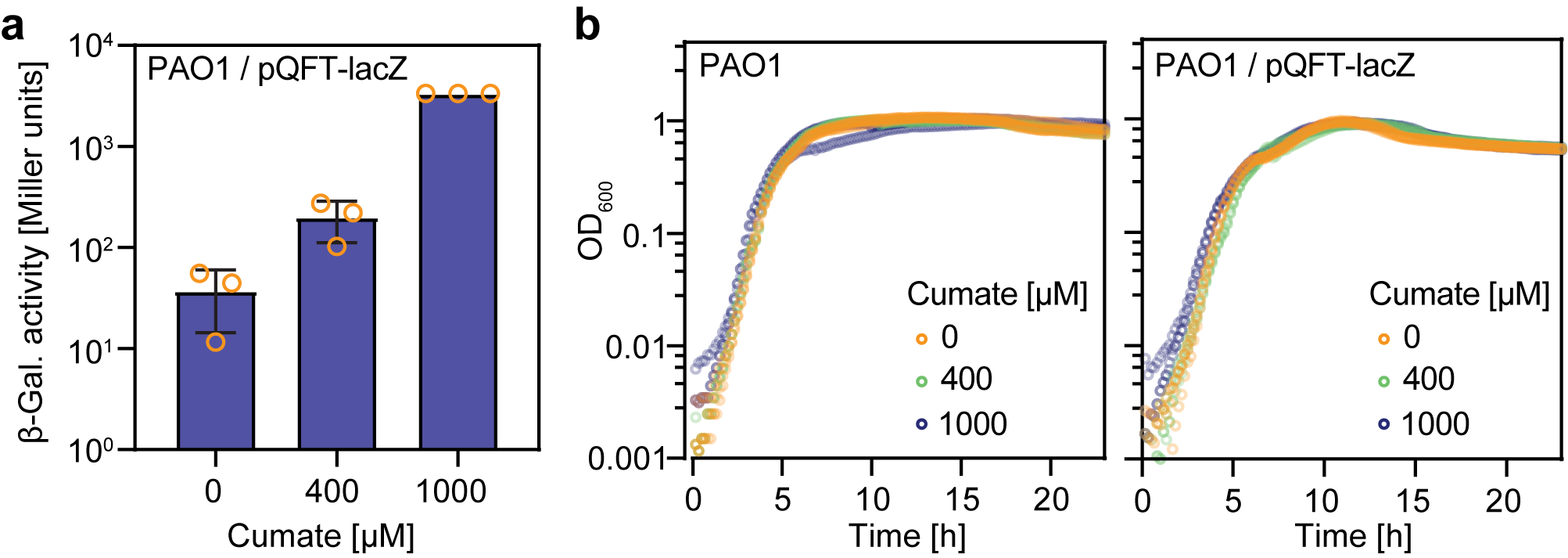

Supplement: Supplemental file 3 — Supplemental material. Download aem.00211-23-s0003.tif, TIF file, 0.7 MB [file aem.00211-23-s0003.tif]

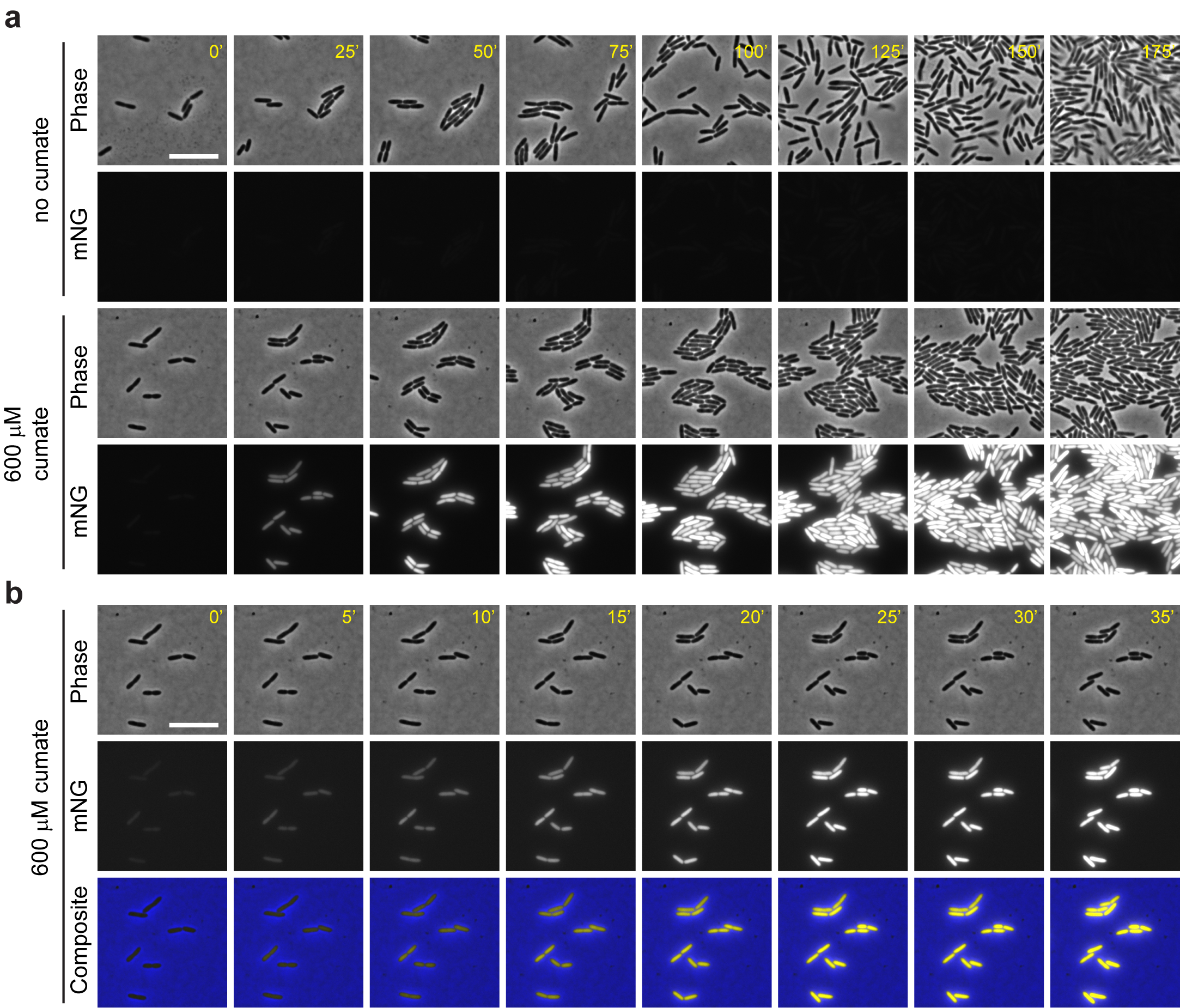

Supplement: Supplemental file 4 — Supplemental material. Download aem.00211-23-s0004.tif, TIF file, 11.9 MB [file aem.00211-23-s0004.tif]

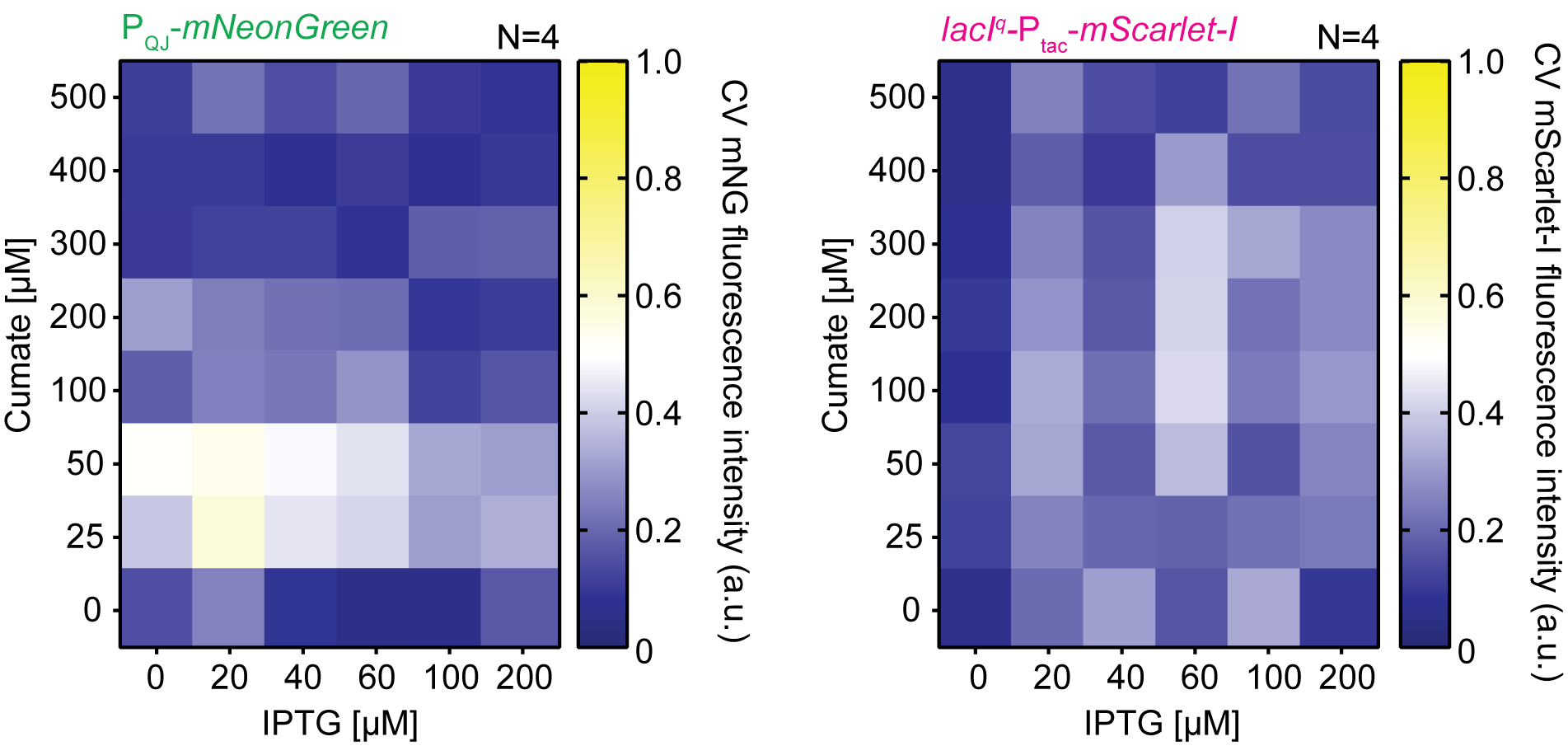

Supplement: Supplemental file 6 — Supplemental material. Download aem.00211-23-s0005.tif, TIF file, 0.7 MB [file aem.00211-23-s0005.tif]

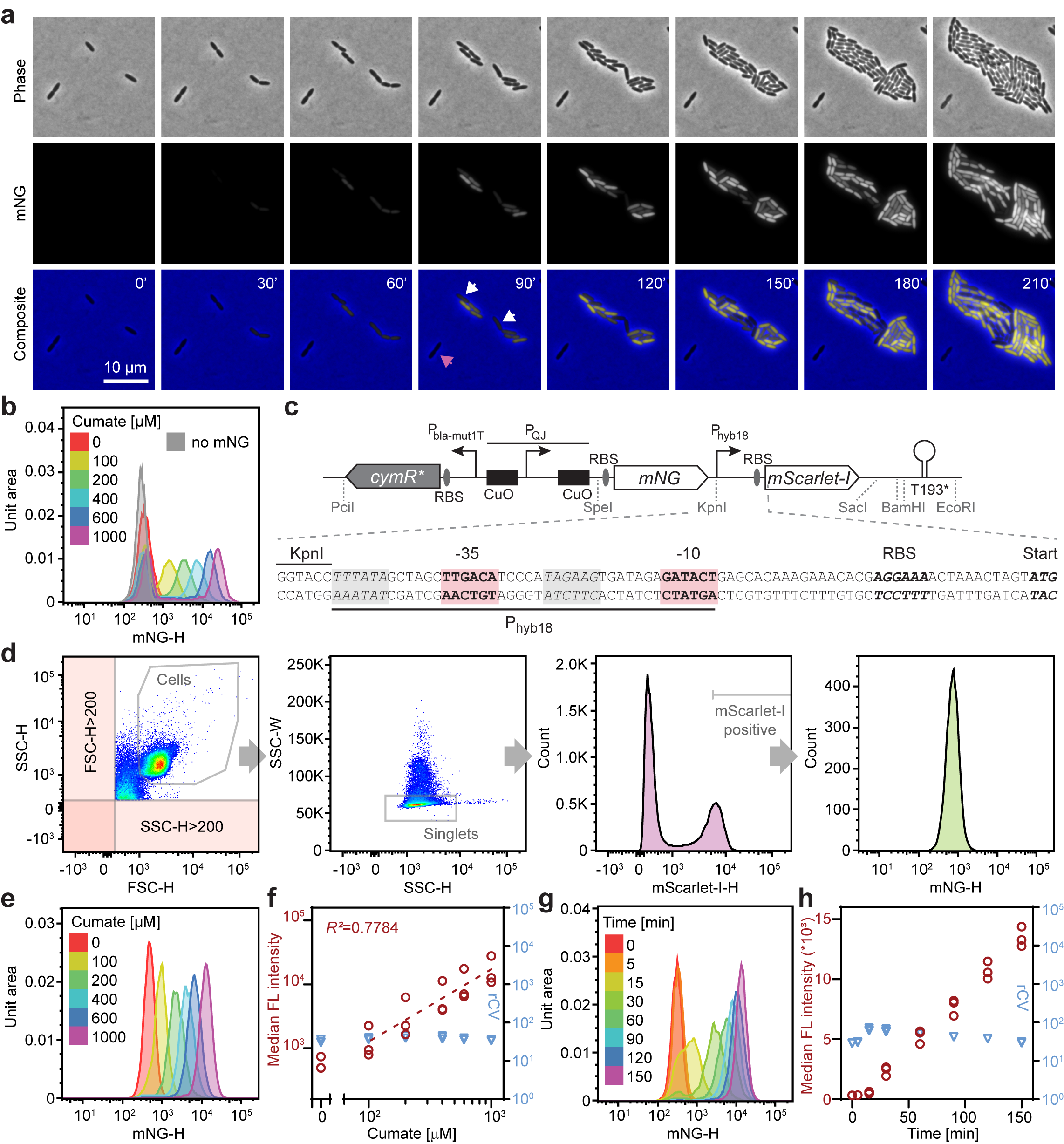

Supplement: Supplemental file 5 — Supplemental material. Download aem.00211-23-s0006.tif, TIF file, 6.4 MB [file aem.00211-23-s0006.tif]
